# Has the Reform of the Japanese Healthcare Provision System Improved the Value in Healthcare? A Cost-Consequence Analysis of Organized Care for Hip Fracture Patients

**DOI:** 10.1371/journal.pone.0133694

**Published:** 2015-07-24

**Authors:** Haruhisa Fukuda, Sayuri Shimizu, Tatsuro Ishizaki

**Affiliations:** 1 Department of Health Care Administration and Management, Graduate School of Medical Sciences, Kyushu University, Fukuoka, Japan; 2 Institute for Health Economics and Policy, Tokyo, Japan; 3 Tokyo Metropolitan Institute of Gerontology, Tokyo, Japan; Department of Transplantation and Renal Medicine, AUSTRALIA

## Abstract

**Objectives:**

To assess the value of organized care by comparing the clinical outcomes and healthcare expenditure between the conventional Japanese “integrated care across specialties within one hospital” mode of providing healthcare and the prospective approach of “organized care across separate facilities within a community”.

**Design:**

Retrospective cohort study.

**Setting:**

Two groups of hospitals were categorized according to healthcare delivery approach: the first group included 3 hospitals autonomously providing integrated care across specialties, and the second group included 4 acute care hospitals and 7 rehabilitative care hospitals providing organized care across separate facilities.

**Participants:**

Patients aged 65 years and above who had undergone hip fracture surgery.

**Measurements:**

Regression models adjusting for patient characteristics and clinical variables were used to investigate the impact of organized care on the improvements to the mobility capability of patients before and after hospitalization and the differences in healthcare resource utilization.

**Results:**

The sample for analysis included 837 hip fracture surgery cases. The proportion of patients with either unchanged or improved mobility capability was not statistically associated with the healthcare delivery approaches. Total adjusted mean healthcare expenditure for integrated care and organized care were US$28,360 (95% confidence interval: 27,787-28,972) and US$21,951 (21,511-22,420), respectively, indicating an average increase of US$6,409 in organized care.

**Conclusion:**

Our cost-consequence analysis underscores the need to further investigate the actual contribution of organized care to the provision of efficient and high-quality healthcare.

## Introduction

Transitional care refers to the set of processes and actions designed to ensure the coordination and continuity of healthcare for patients transferred between different locations or different levels of care within the same location [1]. There has been an increasing level of interest in the costs and effectiveness of transitional care among researchers and policymakers in the US [1–8]. In contrast, Japan’s healthcare provision system in the later part of the 20^th^ century was predominantly based on integrated practice units responsible for providing comprehensive care for a medical condition (and its complications) from the acute to the convalescent phases within a single admission, thereby ensuring continuity of care for patients. This resulted in Japan’s “acute care hospitals” having average length of stay (LOS) durations that are considerably longer than those of other countries in the OECD [9].

The Ministry of Health, Labour and Welfare announced that the direction for Japan’s healthcare provision system in the 21st century would place an emphasis on the provision of organized care siloed by specialties and non-continuous care across separate facilities within a community [10–12]. Here, organized care refers to a delivery system wherein acute care hospitals provide only acute care, and post-acute care is provided in separate and specialized facilities. In other words, the continuum of treatment from acute care to convalescent care prior to social reentry would no longer be provided at individual healthcare institutions, but instead would be consecutively provided by a system of coordinated facilities encompassing different functionalities within a region (this framework is referred to in Japan as *Chiiki Renkei*, or “community-based care”).

In order to bring about effective community-based organized care across healthcare institutions, Japan has introduced community-based care pathways. These pathways refer to the regulated treatment episodes of patients as they progress through the following stages: (1) care pathway from admission to an acute care hospital until discharge, (2) period of transfer to a convalescent care hospital, (3) care pathway from admission to a convalescent care hospital until discharge, and (4) period immediately following discharge from the convalescent care hospital. Community-based care pathways resemble the integrated care pathways (also known as coordinated care pathways, care maps, or anticipated recovery pathways) used in the UK [13], and are regarded as tools intended to encourage interdisciplinary communication, reduce unwanted practice variations, facilitate clinician-patient communication, and improve the systematic collection of clinical data for audit. For these reasons, the Ministry of Health, Labour and Welfare envisioned that these pathways would contribute to the provision of efficient and high quality healthcare, as well as ensure the safety of patients. As a financial incentive for providers to establish community-based care pathways, a supplementary reimbursement system was implemented in 2006. Under this system, hospitals that provide healthcare to patients for hip fractures, cerebrovascular accidents, and malignant tumors are additionally reimbursed for each patient transferred from an acute care hospital to a convalescent care hospital (the acute care hospital is reimbursed US $85 and the convalescent care hospital is reimbursed US $57 per patient).

Evidently, the provision of organized care by specialty across separate facilities within a community is an important policy issue in Japan. However, from the perspective of policymakers, there is insufficient evidence to show the degree of contribution from these policies toward improving patient outcomes or reductions in healthcare resource utilization. The objective of this study was to assess the value of organized care, in which the value of care is defined as patient health outcomes achieved relative to the costs of care [14]. If Japan’s government is working to promote the provision of organized care, this type of care should bring about better health outcomes and/or lower financial costs when compared with traditional integrated care. To this end, this study compares clinical outcomes and healthcare expenditure between the prevailing Japanese “integrated care across specialties within one hospital” mode of providing healthcare and the prospective approach of “organized care by specialty and non-continuous care across separate facilities within a community”. Here, we focus on care provided to older patients admitted for hip fracture, and use a cost-consequence analytical approach by evaluating and presenting health outcomes and costs without separate aggregation of the results. Since the cost-consequence analysis was conducted from the perspective of healthcare payers, costs were evaluated using healthcare expenditure.

## Methods

### Setting

This study was conducted with the cooperation of participant hospitals that could be categorized either to an “integrated care across specialties within one hospital” group or an “organized care within a community” group. Facilities that provided longitudinal care comprising both acute and rehabilitative care within a single facility were designated “integrated care hospitals”. Within the organized care group, facilities that provided acute care with a focus on surgical procedures were designated “acute care hospitals”, while facilities that provided post-surgical care mostly concerned with the social reintegration of patients were designated “rehabilitation hospitals”. The selection of participant hospitals for this study first involved the identification of acute care hospitals with management staff who demonstrated interest in regional collaboration at academic meetings and conferences. Next, rehabilitation hospitals with many transferred cases from the aforementioned acute care hospitals were identified.

The integrated care group comprised 3 hospitals (designated Hospitals A, B, and C), and the organized care group comprised 4 acute care hospitals (Hospital D, F, I, and L) and 7 rehabilitation hospitals (Hospital E, G, H, J, K, M, and B). One rehabilitation hospital (Hospital B) within the organized care group also provided integrated care from the acute to rehabilitation stages. The structural characteristics of the participant hospitals are presented in Table 1.

**Table 1:** Structural characteristics of participant hospitals.

Hospital ID^a^	No. of beds (acute / rehabilitation)	No. of physicians	No. of nurses	Patient volume per day	Overall LOS	Population density^b^ (person / km^2^)
A^1^	315 / 0	34.1	242.0	294.2	17.4	500
B^2^	43 / 123	13.6	83.0	39.0 [79.0]	12.2 [51.5]	8,100
C^1^	502 / 0	141.1	489.9	475.0	12.4	250
D^1^	456 / 0	131.3	568.0	398.7	12.7	500
E^1^	27 / 90	8.1	61.6	[89.1]	[64.5]	500
F^3^	400 / 0	156.5	643.1	387.9	9.4	1,900
G^3^	80 / 66	16.4	75.6	[67.1]	[105.7]	1,900
H^3^	42 / 21	8.7	17.5	[20.9]	[37.7]	1,900
I^2^	592 / 0	181.7	649.4	517.2	13.1	1,100
J^2^	66 / 42	8.3	61.5	[36.8]	[169.1]	1,100
K^2^	169 / 30	20.9	126.8	[29.7]	[236.9]	1,100
L^2^	230 / 0	61.0	194.2	211.1	13.4	8,100
M^2^	0 / 596	20.0	127.0	[484.3]	[198.1]	6,800

^a^ Data indicated with 1, 2, and 3 for the number of beds, physicians, and nurses were from 2014, 2015, and 2012, respectively; data for patient volume and overall LOS were from 2013, 2014, and 2011, respectively.

^b^ Population density of the city (correct at February 2015) where each hospital is located.

LOS, length of stay

### Patients

The subjects for analysis were patients aged 65 years and above who had undergone surgery for hip fracture in the participant hospitals. In the integrated care group, patients discharged to their homes were selected for inclusion, whereas patients transferred to another hospital were excluded from analysis. In the organized care group, patients were selected for analysis if they had been transferred from a participant acute care hospital to a participant rehabilitation hospital; patients discharged to their homes or to a non-participant rehabilitation hospital were excluded from analysis.

### Data

The input of healthcare resources was evaluated using LOS and healthcare expenditure; the analysis was conducted from the perspective of healthcare payers, and the aforementioned variables were measured in days and US dollars (1 US $ = 105.89 yen, derived from purchasing power parities at 2012), respectively.

Information on healthcare resources was obtained using administrative claims data. In 2003, Japan implemented a new hospital reimbursement system known as the Diagnosis Procedure Combination (DPC) system, which uses a per-diem payment system (PDPS). All acute care hospitals participating in this study had implemented this system prior to analysis, and we collected these DPC claims data. However, the rehabilitation hospitals in this study do not use a DPC/PDPS system, but instead submit claims for reimbursement under the conventional Japanese fee-for-service (FFS) system. The corresponding paper-based claims data under the FFS system were also collected for analysis. For the integrated care group, 2 of the hospitals utilized DPC data, while the third hospital provided paper-based claims data.

The amount of healthcare resources utilized in the organized care hospitals was calculated using the accumulative healthcare expenditure and LOS from admission to an acute care hospital until discharge from a rehabilitation hospital. Because there are no unique patient identification numbers used across healthcare providers in Japan, claims data from the acute and rehabilitation hospitals were merged using patient birthdates, sex, and admission/discharge dates. Healthcare resources utilized in integrated care hospitals were calculated as the total healthcare expenditure and LOS from admission to discharge within a single hospitalization episode.

The differences in healthcare resource utilization between the organized care and integrated care groups were analyzed using an exposure variable involving hospital pathways; in this binary variable, hospitals that provided integrated care were assigned a value of 0, while hospitals that provided organized care were assigned a value of 1.

As differences in patient case mix can substantially influence the amount of healthcare resources utilized, patients were adjusted for the following covariates: age, sex, fracture location, surgical method, residence prior to injury, mobility capability prior to injury, mobility capability at time of discharge, long-term care needs level, and year of admission. Long-term care needs level is an indicator that incorporates factors such as physical function, vital function, cognitive function, and functional independence.

### Statistical Analysis

To evaluate the effectiveness of organized care, we utilized a multivariable logistic regression model to investigate the clinical outcome of whether patients experienced “improvements or no changes in mobility capability” or “reductions in mobility capability” between the pre-injury and post-discharge stages. Therefore, we excluded patients whose mobility capability prior to injury was unknown.

In order to analyze the differences in healthcare resource utilization while accounting for patient characteristics and clinical outcomes, a multivariable regression model was used to adjust for the effects of the covariates. In this analysis, cases that fell beyond the lower and upper 2.5% of the total LOS duration were excluded in order to remove the disproportionate influence from these outliers. We analyzed the data using a Generalized Linear Model (GLM) with a log-link function and a gamma distribution. Regression coefficients of the GLM were reconverted to the original units of healthcare resources (Days and US $) using the recycled prediction method. Finally, 95% confidence intervals were estimated using bootstrapping.

All statistical analyses were conducted using Stata 13.1 for Windows (Stata Corp., College Station, TX, USA)

### Ethics Statement

This study was approved by the Ethics Committee of the Institute for Health Economics and Policy. The study was conducted with a waiver of patient consent. Patient records/information was anonymized and de-identified prior to analysis. The study complied with the Ethical Guidelines for Epidemiological Research of the Japanese national government, which include guidelines on protecting patient anonymity, and all the necessary conditions were satisfied for informed consent to be waived.

## Results

### Healthcare Resource Utilization

The final sample for analysis consisted of 837 cases. A summary of the healthcare resources used by the patients admitted to the participant hospitals for femoral neck fracture surgery is presented in Table 2. In organized acute care Hospitals I and L, pre-surgical LOS was the same regardless of the subsequent rehabilitation hospital for rehabilitative care. For post-surgical LOS durations in organized acute care hospitals, Hospitals F and I showed similar durations regardless of subsequent rehabilitation hospitals (11 and12 days in Hospital F and 18 and 20 days in Hospital I).

**Table 2:** Healthcare resource utilization characteristics by hospital.

Type of care	Integrated care			Organized care						
Acute care hospital ID	A	B	C	D	F		I		L	
Rehabilitation hospital ID				E	G	H	J	K	M	B
Study period	Apr 2008–Dec 2010	Dec 2006–Mar 2011	2008–2009 (Jul–Dec)	Apr 2008–Dec 2010	Mar 2010–Dec 2010	Sep 2009–Dec 2010	2008–2010 (Jul–Dec)	2008–2010 (Jul–Dec)	2006–2010 (Jul–Dec)	2006 Dec–2011 Mar
Pre-surgical LOS	1 (1–2)	4 (3–6)	2 (1–4)	4 (2–6)	3 (3–6)	2 (1–3)	3 (2–5)	3 (2–5)	4 (2–6)	4 (3–4.5)
Post-surgical LOS	n.a.	n.a.	n.a.	15 (13–20)	12 (10–16)	11 (9–12)	20 (16–29)	18 (15–22)	17 (15–24)	25.5 (22.5–29)
LOS at rehabilitation hospital	n.a.	n.a.	n.a.	54 (31–81)	62 (33–71)	38 (29–50)	71 (44.5–95)	70.5 (64–78)	83 (57–88)	71 (40–86.5)
Total LOS	46 (27–70)	73.5 (50–96)	23.5 (19–28)	77 (53–98)	76 (53–95)	54 (45–61)	100 (79–115)	90 (80–103)	106 (82–115)	101 (66–120.5)
Expenditure at rehabilitation hospital (US$)	n.a.	n.a.	n.a.	13806 (7645–21499)	14969 (9528–21003)	9556 (7836–12760)	14938 (8452–18657)	15820 (13199–19256)	21126 (13942–23607)	20227 (12694–25109)
Total expenditure (US$)	19414(15026–24750)	31366 (23836–38624)	13001 (10821–17371)	27304 (20125–34767)	30206 (21525–34822)	20397 (18718–26387)	29519 (22607–34484)	28883 (21527–33248)	34654 (28633–39701)	34018 (26882–38678)

LOS, length of stay; Values are presented as “median (25^th^ percentile– 75^th^ percentile)”

Hospitals J and K are both rehabilitation hospitals that provide organized care within the same region. Although the median LOS durations of these 2 hospitals were similar at 71 days and 70.5 days, respectively, the LOS distributions were different. For other rehabilitation hospitals within the same regions, both the median LOS durations and distributions were markedly different. This trend was also observed for healthcare expenditure.

The total amounts of healthcare resources used were higher in the 25th and 75th percentiles for the acute care and rehabilitation hospitals belonging to the organized care group when compared with integrated care hospitals.

### Patient Characteristics

The patient characteristics of the study sample are shown in Table 3. Femoral neck fractures accounted for 53.6% of the study sample, whereas intertrochanteric fractures accounted for 42.9%. The data revealed different proportions of hip fracture types in different acute care hospitals. In surgical methods, all facilities showed a higher proportion of osteosynthesis relative to femoral head prosthesis.

**Table 3:** Distribution of patient characteristics by hospital.

Type of care	Integrated care			Organized care							Overall
Acute care hospital ID	A	B	C	D	F		I		L		
Rehabilitation hospital ID				E	G	H	J	K	M	B	
Number of cases	(n = 181)	(n = 205)	(n = 198)	(n = 57)	(n = 25)	(n = 66)	(n = 20)	(n = 30)	(n = 39)	(n = 16)	(n = 837)
Age											
85-	37.0	35.1	53.0	47.4	56.0	42.4	45.0	43.3	35.9	37.5	42.4
Sex											
Female	86.2	82.0	88.4	77.2	88.0	86.4	85.0	86.7	82.1	87.5	84.9
Fracture location											
Neck	56.4	52.7	53.5	56.1	40.0	33.3	85.0	90.0	51.3	31.3	53.6
Trochanter	39.2	41.0	44.4	38.6	56.0	66.7	15.0	10.0	48.7	68.8	42.9
Others	4.4	6.3	2.0	5.3	4.0	0.0	0.0	0.0	0.0	0.0	3.5
Surgical method											
Osteosynthesis	61.3	50.7	70.2	61.4	60.0	68.2	50.0	60.0	53.8	68.8	60.8
Head prosthesis	36.5	46.3	29.8	26.3	40.0	27.3	50.0	26.7	46.2	31.3	36.3
Others	2.2	2.9	0.0	12.3	0.0	4.5	0.0	13.3	0.0	0.0	2.9
Residence prior to injury											
Home	74.6	26.8	0.0	82.5	88.0	59.1	80.0	83.3	0.0	43.8	41.3
NOT home	22.7	13.2	0.0	15.8	12.0	34.8	15.0	16.7	0.0	12.5	13.5
Unknown	2.8	60.0	100	1.8	0.0	6.1	5.0	0.0	100	43.8	45.2
Mobility capability prior to injury											
Self-reliance	43.1	41.0	0.0	50.9	60.0	47.0	50.0	53.3	56.4	50.0	35.0
Walking aid ^a^	31.5	13.7	0.0	43.9	28.0	39.4	45.0	46.7	17.9	31.3	21.3
Wheelchair ^b^	11.0	6.8	0.0	3.5	12.0	13.6	5.0	0.0	10.3	0.0	6.3
Unknown	14.4	38.5	100	1.8	0.0	0.0	0.0	0.0	15.4	18.8	37.4
Mobility capability at time of discharge											
Self-reliance	15.5	18.0	0.0	28.1	16.0	4.5	15.0	6.7	0.0	12.5	11.4
Walking aid ^a^	37.6	30.2	0.0	47.4	40.0	33.3	45.0	50.0	0.0	31.3	26.0
Wheelchair ^b^	33.1	27.3	0.0	10.5	16.0	33.3	40.0	40.0	0.0	25.0	20.5
Unknown	13.8	24.4	100	14	28.0	28.8	0.0	3.3	100	31.3	42.1
Changes in mobility capability from pre-injury to post-discharge ^c^											
1 level up	2.2	0.5	0.0	12.3	0.0	1.5	5.0	3.3	0.0	0.0	1.8
No change	40.9	32.7	0.0	42.1	40.0	27.3	40.0	20.0	0.0	25.0	25.2
1 level down	30.9	17.1	0.0	28.1	32.0	28.8	35.0	56.7	0.0	31.3	19.5
2 level down	9.9	7.8	0.0	3.5	0.0	13.6	20.0	16.7	0.0	6.3	6.6
Unknown	16.0	42.0	100	14.0	28.0	28.8	0.0	3.3	100	37.5	47.0
Long-term care needs levels											
Non-qualified	1.7	13.2	0.0	56.1	8.0	25.8	15.0	16.7	0.0	18.8	11.0
Application process/Required assistance	7.2	14.2	0.0	1.8	0.0	6.0	35.0	20.0	0.0	25.0	7.6
Classes 1–3	21	47.4	0.0	22.8	36.0	36.4	25.0	43.4	0.0	43.9	24.6
Classes 4–5	3.3	10.8	0.0	3.5	12.0	6.1	0.0	3.3	0.0	0.0	4.5
Unknown	66.9	14.6	100	15.8	44.0	25.8	25.0	16.7	100	12.5	52.2
Admission year											
2006	0.0	3.4	0.0	0.0	0.0	0.0	0.0	0.0	2.6	25.0	1.4
2007	0.0	18.0	0.0	0.0	0.0	22.7	0.0	0.0	2.6	12.5	6.6
2008	45.3	22.9	46	36.8	0.0	27.3	25.0	36.7	5.1	18.8	33.5
2009	26.5	26.8	54	56.1	8.0	33.3	50.0	36.7	56.4	43.8	37.8
2010	28.2	29.3	0.0	7.0	92.0	16.7	25.0	26.7	33.3	0.0	20.9

Values are presented as “proportion (%)”. Cases with missing data were included in the “unknown” category.

^a^ “walking aid” includes canes, walkers, and wheeled walking aids;

^b^ “wheelchair” includes wheelchairs and bedridden patients;

^c^ Proportions were calculated with the denominator comprising cases with available data for mobility capability prior to injury and at the time of discharge.

It was difficult to discern any inter-hospital trends with regard to mobility capability prior to injury, mobility capability at time of discharge, and long-term care needs level. When limiting the analysis only to hospitals with comparable data, approximately half of the patients were able to walk independently prior to injury, whereas another 40–50% relied on walking aids. With regard to mobility capability at the time of discharge, approximately 10% of the patients were able to walk independently, and similar proportions of patients required the use of walking aids and wheelchairs.

### Effectiveness Analysis

The changes in mobility capability prior to injury and at discharge are also presented in Table 3. A designation of “1 level up” indicates an improvement of one stage; for example, patients who required the use of a walking aid and patients who required a wheelchair prior to injury would be discharged with the ability to walk independently and with a walking aid, respectively. When comparing the changes in mobility capability between the integrated care group and the organized care group, the latter did not show any significantly higher proportion of cases with improved or unchanged mobility capability (P = 0.117, chi-squared test). Multivariate logistic regression analyses also showed that neither of the 2 approaches of healthcare delivery was statistically associated with improvements in mobility capability after adjusting for covariates (Table 4).

**Table 4:** Results of logistic regression analysis for mobility capability and generalized linear regression analysis for healthcare resource utilization.

Variables		Response variables					
		Improvements/no change in mobility capability		Total length of stay		Total expenditure	
		Odds Ratio	P-value	Multiplicative Effect	P-value	Multiplicative Effect	P-value
Age	1 year	1.013	0.395	1.00	0.293	1.00	0.093
Sex (Reference: Male)							
Female		1.755	0.088	1.02	0.687	1.01	0.700
Type of care (Reference: Integrated care)							
Organized care		0.801	0.393	1.59	0.001	1.29	0.013
Fracture location (Reference: Neck)							
Trochanter		0.752	0.238	1.00	0.950	1.06	0.084
Others		0.399	0.004	1.40	0.019	1.37	< 0.001
Surgical method (Reference: Osteosynthesis)							
Head prosthesis		1.143	0.603	1.05	0.448	1.35	< 0.001
Others		1.167	0.724	0.90	0.325	1.02	0.782
Residence prior to injury (Reference: Home)							
NOT home		1.301	0.052	0.72	0.001	0.84	0.002
Unknown		2.275	< 0.001	1.05	0.501	1.13	0.029
Mobility capability prior to injury (Reference: Self-reliance)							
Walking aid		-	-	1.01	0.843	1.00	0.985
Wheelchair		-	-	0.82	< 0.001	0.89	0.005
Unknown		-	-	0.77	0.084	0.84	0.141
Mobility capability at time of discharge (Reference: Self-reliance)							
Walking aid		-	-	1.19	0.008	1.14	< 0.001
Wheelchair		-	-	1.20	0.180	1.13	0.151
Unknown		-	-	0.88	0.261	0.90	0.140
Long-term care needs levels (Reference: Non-qualified)							
Application process/Required assistance		0.861	0.511	1.41	< 0.001	1.26	< 0.001
Classes 1–3		0.955	0.811	1.64	< 0.001	1.42	< 0.001
Classes 4–5		2.276	0.037	1.68	< 0.001	1.53	< 0.001
Unknown		0.828	0.507	1.17	0.045	1.06	0.411
Admission year (Reference: 2010)							
2006		N.A.		1.56	0.001	1.21	0.188
2007		0.225	0.008	1.26	0.232	1.08	0.564
2008		0.793	0.203	0.97	0.727	0.90	0.102
2009		1.245	0.492	0.91	0.280	0.87	0.023

### Cost Analysis

Table 4 also shows the results of the GLM analyses that used total LOS and healthcare expenditure as response variables. When compared with integrated care, organized care was found to be significantly associated with prolonged LOS (P = 0.001) and increased healthcare expenditure (P = 0.013). Other covariates that showed significant association with healthcare resource utilization were fracture location, surgical method (only for total healthcare expenditure), residence prior to injury, mobility capability prior to injury, mobility capability at time of discharge, long-term care needs level, and admission year.

As the results presented in Table 4 are estimated multiplicative effects of GLMs, we reconverted these effects to the actual values for LOS (days) and healthcare expenditure (US$), which are presented in Table 5. These estimated results reflect adjustments for patient characteristics and clinical outcomes. The total LOS durations for integrated care and organized care were 50.1 days (95% confidence interval: 48.9–541.5) and 79.9 days (78.1–82.0), respectively, indicating an average increase of 29.8 days in organized care. The average total expenditure for organized care was US$28,360 (27,787–28,972), which was substantially higher than integrated care (US$21,951; 21,511–22,420).

**Table 5:** Comparisons of adjusted resource utilization by type of care and hospital.

Type of care^a)^		Total LOS, days (95% CI)	Total Expenditure, $US (95% CI)
Hospital ID ^b)^			
**Integrated care**		50.1 (48.9–51.5)	21,951 (21,511–22,420)
A		47.1 (46.5–47.7)	18,610 (18,344–18,844)
B		70.8 (69.9–71.9)	30,267 (29,881–30,663)
C		27.5 (27.2–27.9)	15,599 (15,401–15,794)
**Organized care**		79.9 (78.1–82.0)	28,360 (27,787–28,972)
D	E	81.2 (80.1–82.2)	29,132 (28,716–29,496)
F	G	71.6 (70.7–72.5)	25,585 (25,246–25,915)
	H	62.4 (61.7–63.2)	24,353 (24,062–24,682)
I	J	90.7 (89.7–91.9)	25,787 (25,484–26,111)
	K	79.6 (78.5–80.7)	25,659 (25,366–26,009)
L	M	106.6 (105.2–108)	36,029 (35,565–36,506)
	B	88.7 (87.5–89.8)	33,307 (32,893–33,712)

^a)^ Calculated from the regression coefficients for the “type of care” exposure variable in the generalized linear model shown in Table 4.

^b)^ Calculated from the regression coefficients for the “hospital pathway” exposure variable in a generalized linear model (coefficient data not shown). Other covariates were the same as those presented in Table 4.

With respect to hospital pathway, Hospitals A and C (belonging to the integrated care group) showed substantially shorter total LOS durations than organized care hospitals. However, Hospital B, which also belonged to the integrated care group, had longer total LOS durations than the regional collaborative combinations of Hospitals F and G and Hospitals F and H. The shortest total LOS duration among the hospitals was 27.5 days, which was observed in Hospital C—an integrated care provider. The longest LOS duration was 106.6 days, observed in the regional collaborative combination of Hospitals L and M. This indicates a maximum difference of 79.1 days in LOS at the hospital level. Furthermore, large variations were observed when comparing the total LOS durations between different rehabilitation hospitals (Hospital G vs. Hospital H, Hospital J vs. Hospital K, and Hospital M vs. Hospital B) for patients who had been transferred from the same acute care hospital. The differences in LOS durations for different rehabilitation hospitals were 9.2 days (Hospital G vs. Hospital H), 11.1 days (Hospital J vs. Hospital K), and 17.9 days (Hospital M vs. Hospital B). Similar patterns were observed in healthcare expenditure. Hospital B (which provided integrated care) was shown to have higher total healthcare expenditure than organized care hospitals, except for the regional collaborative combinations of Hospital L with Hospitals M or B. The lowest healthcare expenditure was observed in Hospital C (comprehensive care) at US $15,599, whereas the highest healthcare expenditure was observed in the regional collaborative combination of Hospitals L and M, at US $36,029. There was a maximum difference between the healthcare provision approaches of US $20,430 at the hospital level.

## Discussion

The incentive system to encourage organized care in Japan was implemented in 2006 without a strong evidence-based foundation. The underlying premise for the introduction of this system was based on a case report from Kumamoto City [4], which conceptualized that the implementation of organized care for acute care hospitals and rehabilitative care hospitals—ostensibly more adept at providing specialized care in their respective fields—would result in the concentrated investment of healthcare resources, thereby allowing the provision of “efficient and high quality” healthcare. The incentive system was implemented in lieu of scientific evidence proving that organized care hospitals provide more efficient and higher quality care than hospitals that have heretofore provided integrated care.

In this study, the comparison of resource utilization (total LOS and healthcare expenditure) and clinical consequence (improvements in mobility capability) between organized care hospitals and conventional integrated care hospitals revealed that the provision of organized care has yet to demonstrate higher quality of care. Patients who were residing in locations other than their homes prior to injury were generally shown to have shorter LOS durations, suggesting that specialists in rehabilitation hospitals may have determined that patients can be discharged relatively earlier to a care facility (rather than to patient residences) even if the patients were not completely rehabilitated, as care facilities would be able to provide further rehabilitative care. Alternatively, it is possible that patients who are discharged to their homes may have delayed discharges due to a need for appropriate modifications to their homes to support their reduced mobility.

Since 2001, the provision of organized care siloed by specialties and non-continuous care across separate facilities within a community has continued to be an indispensable component of discussions on the healthcare provision system in Japan. On February 17, 2012, the Cabinet-approved “Comprehensive Reform of Social Security and Tax” [15] reiterated that future reviews of healthcare and nursing should include “the functional differentiation and enhancement of hospitals and hospital beds, including clarifying the position of acute care hospital beds and functional enhancements resulting from the concentrated investment of healthcare resources”. Researchers have previously reported a positive association between the number of procedures and post-surgical outcomes in orthopedic surgery [16], and this may support the expectations that concentrating acute care services to acute care hospitals would improve efficiency and quality. Our cost-consequence analysis revealed that acute care hospitals that provide care as part of organized care groups had relatively standardized levels of resource utilization, and that a certain level of functional enhancement of acute care hospital beds could therefore be achieved. However, large variations were observed in the rehabilitation hospitals, even among those that received patients from the same acute care hospital. This indicates that the lack of standardization stems not from the acute care hospitals, but from the rehabilitation hospitals.

Both of the 2 integrated care hospitals that exhibited low healthcare resource utilization were PDPS-implemented hospitals, whereas the non-PDPS (i.e. FFS) comprehensive care hospital (Hospital B) showed higher healthcare resource utilization. However, we consider that the difference in payment system had a limited effect on resource utilization. The newly implemented PDPS was in a transition phase, and the amount of reimbursement to PDPS-implemented hospitals was politically designed to be equivalent to that of FFS-implemented hospitals for the provision of the same services to similar patients. The observed difference in healthcare resource utilization, therefore, was perhaps influenced in part by the standardized format of healthcare and clinical data required in DPC-implemented hospitals. Policies to reform rehabilitation hospitals should therefore first encompass support strategies for upgrading the collection and maintenance of data.

Since healthcare delivery in the US is essentially functionally differentiated, researchers [1–8] have focused on the methods of transferring patients during transitional care. On the other hand, the introduction of organized care delivery in Japan is much more recent. This gives Japan more time to judiciously evaluate the costs and effectiveness of organized care in the Japanese healthcare setting, and to properly inform the policymaking process. To the best of our knowledge, this study is the first empirical analysis of the value of organized care for older patients with hip fracture in Japan.

As there are several limitations to this study, the findings should be interpreted with caution. First, there was a limitation in acquiring patient clinical data. As indicated by our findings, patient characteristics such as residence prior to injury, mobility capability prior to injury, mobility capability at time of discharge, and long-term care needs level were significantly associated with the amount of healthcare resources utilized. Although it was possible to conduct a chart review analysis in all but one of the acute care hospitals in our study, there were numerous cases in which the aforementioned data had not been recorded. Second, there were limitations to the availability of data concerning patient characteristics. The following indicators are unevenly collected in many of Japan’s rehabilitative healthcare facilities: Degree of independence for daily living for disabled older people, degree of independence for daily living for older people with dementia, the Hasegawa Dementia Scale, the Functional Independence Measure, and the Barthel Index. Although the use of different indicators by different facilities precluded the utilization of these factors in a multi-institutional analysis, we consider that the variations in physical function, vital function, cognitive function, and functional independence among patients were partially adjusted by the inclusion of the long-term care needs level indicator. Third, the participant hospitals may not be representative of all hospitals in Japan due to the sample selection methods and the small sample size. In the organized care group, we selected participant acute care hospitals whose management staff demonstrated interest in regional collaboration at relevant academic meetings. Therefore, the healthcare resource utilization of the organized care group in this study would likely have a degree of bias toward smaller estimates when compared with all organized care hospitals in Japan. Also, Japan has approximately 8,500 hospitals located throughout the country, and this study only includes 13 of these hospitals. Therefore, it is difficult to directly extrapolate our conclusions to Japanese health policy. However, Japan does not currently have a large, publicly accessible cohort database that includes data on expenditure, patient clinical characteristics, clinical outcomes, and hospital pathways. Fourth, we used patients discharged to their homes for the integrated care group while using patients transferred from an acute care hospital to a rehabilitation hospital for the organized care group. The non-inclusion of patients discharged from an acute care hospital to their homes for the organized care group may inadvertently exclude patients whose activities of daily living before admission were too poor to expect substantial recovery (wheelchair and bedridden), thereby leading to the possible introduction of bias into the study. However, the proportion of patients discharged from acute care hospitals to their homes was relatively small (ranging from 0.8% to 15.4%) among the hospitals of the organized care group. We conducted additional multivariable regression analyses (results not shown) that excluded patients whose mobility capability prior to injury was unknown or poor; when compared with our original analysis, the results of these additional analyses showed no major changes in the effects of type of care on mobility capability (odds ratio and p-value were 0.979 and 0.942, respectively), total LOS (coefficient and p-value were 0.275 and 0.007, respectively), and total expenditure (coefficient and p-value were 0.143 and 0.120, respectively).

Despite these limitations, our study has several policy implications. The first is the demonstration of the variations in healthcare resource utilization by rehabilitation hospitals in the organized care group. This may indicate a non-optimal payment system and a lack of standardization in clinical processes in rehabilitation hospitals. Because rehabilitation hospitals are reimbursed using a FFS system and the upper limits for LOS are high, the best management strategy for rehabilitation hospitals is to fill their hospitals beds within the stipulated maximum limits of LOS. Therefore, if a new prospective payment system or reductions to the upper limits for LOS are implemented, the overly long stays observed in this study may be reduced or even eliminated. It is also important to note that large variations were observed in the rehabilitation hospitals, even among those that received patients from the same acute care hospital. Although the implementation of community-based care pathways may include a tendency to focus on reductions of redundant services such as diagnostic tests or polypharmacy, the large variations in LOS durations in rehabilitation hospitals warrant further attention. The drive to organized care should place a high priority on improving payment system in rehabilitation hospitals, in addition to standardizing care and resource utilization.

Secondly, despite the recent emphasis on organized care in Japan’s healthcare provision system, the lack of a means to evaluate the performance of such a policy should be recognized as a major flaw in the system. For example, the provision of economic incentives for community-based care pathways should involve mechanisms that allow for policy evaluation, such as the establishment and compulsory reporting of standardized factors involving healthcare resource utilization, clinical outcomes, and patient factors. Failure to include an evaluative component of the system would hinder the assessment of the “efficient and high quality healthcare” that the Ministry of Health, Labour and Welfare aspires to achieve, ultimately resulting in the loss of opportunities to improve policy.

## Conclusion

After adjusting for patient characteristics, comparisons in healthcare resource utilization (assessed using the total costs of the full cycle of care for each patient) and improvements in patient outcomes between organized care hospitals and integrated care hospitals showed that the latter generally used less healthcare resources, despite having similar outcomes. Despite possible limitations in the generalizability of our findings, our study underscores the need for further research to investigate whether organized care actually contributes to the provision of efficient and higher quality healthcare.
